# Items, News and Gossip

**Published:** 1872-04

**Authors:** 


### Items, News and Gossip.

[We are indebted to "*The Doctor*," (London, Eng.) for the following items.—ED.]

In the *Riv. Clin. di Bologna*, February, 1872, Dr. Bonfigli mentions that Jones recommends belladonna in gradually increasing doses in spermatorrhœa. Atropine is recommended by Hosch in many cases of myopia. Philipson recommends belladonna in constipation.—Echeverria and MacDonald recommend conium as the best narcotic remedy for epileptics. The juice of the fresh plant is used.—Mr. Veagh considers datura tatulæ the best remedy for asthma, far better than stramonium.—Dr. Reichard, of Riga, has found chloral hydrate of great use in cholera; four grammes of chloral produced sleep in a few minutes, and the patient recovered. —Dr. Lutz has found great benefit from the use of bromide of potassium in epilepsy. His doses are large, from 10 to 20 grammes daily. No great inconvenience has been produced, except some few papules on the skin. In one case of nocturnal incontinence of urine, it was very useful. Dr. Northrop says that a gramme of this salt every hour will get rid of tape-worm.—Dr. Palmberg has used ergot of rye with success in three cases of chronic diarrhœa. To children he gave 50 centigramme doses of the ergot in watery extract in the day; to adults he gives 1.20 grammes.—Dr. Vance alleges that he has obtained cures in some cases of palsy by the subcutaneous injection of strychnine, in the dose of five millegrammes of the phosphate.——In the *Med.-Chir. Centralbl.*, 1871, Dr. Hemard asserts that for the last twenty years he has obtained the cure of soft and hard sores of venereal ulcers by simply irrigating the parts with cold water. A vessel of cold water is fixed to the walls of the room, at such a height that the water which comes from it through a tube, attains a certain force of projection. The patient has no more to do but to wash his ulcers every three or four hours under this stream; in a few days the ulcer becomes clean, and quickly heals. All other treatment is superfluous. In ulcers of the prepuce, which are out of sight, after irrigation, a little starch flour is introduced. When the superficies of the ulcer has lost its characteristic aspect, a stratum of collodion is painted on it, and it soon heals. —— Dr. Poggi, writing to Dr. Scarenzio (*Giornale dell. Mall. del. Pel.*), says that in his wards a man, C. V., soldier in a cavalry regiment, entered in August, 1870, with syphilitic ulcerations and mucous patches in the fauces. Three calomel injections were practiced with the syringe; the first on 26th August; the second on 5th September. An abscess succeeded to each injection, which opened spontaneously; but the cure was complete on the 6th December.—P. V., a soldier, in the same regiment, entered hospital the 18th November, 1870, for mucous papules (some months after being attacked with indurated sore on the penis). Injection of calomel was used on the 26th November. An abscess after this was opened 6th December. The mucous papules disappeared without any further treatment, and he left completely cured in January, 1871.—C. A., a soldier in the same regiment, entered hospital 29th November, 1870, for scattered papular syphilis over the whole body. The first injection was made on December 1st. The abscess formed on the 6th day, and as it was very painful (indeed, an exceptional affair), it was opened on the 8th. This, too, had the appearance of sanguineous abscess. He got perfectly well without any other treatment and left hospital 4th January, 1871. In three cases the dose of calomel was 25 centigrammes (about four grains), and the injection was made at the external and posterior aspect of the arm, and external aspect of the thigh.—T. P., a soldier, entered hospital for indurated ulcers on the glands and inguinal buboes, 24th August, 1869. The bubo suppurated, and then followed sinuous abscesses which required numerous incisions. Injections of calomel suspended in mucilage of gum arabic were made use of in doses of 30 centigrammes (5 grains). The first on the 3rd October, 1869; the second on the 7th October; and the third on the 22nd of the same month. He left completely cured on the 26th March, 1870. The long duration of treatment was caused by the loss of substance in the soft parts. In this case the abscesses were all open, and washed with solution of sublimate, 15 centigrammes to 300 grammes of distilled water. —— In the *Pabellon Medico* a case of scarification of the os uteri is mentioned where Dr. Cortejarena succeeded in notably alleviating symptoms of congestion of the cervix uteri by scarifying the cervix, instead of applying leeches. We notice that Lister's plan of dressing wounds is in favor with Spanish surgeons. —— Prof. Bolkin, in the cholera epidemic which took place in St. Petersburgh in 1871, made use of the sulphate of quinine, which he administered in doses of 20 to 30 centigrammes at a time, three or four times a day, and even more frequently if the substance was vomited; in many cases he also made use of a subcutaneous injection of the remedy. After using this remedy the mortality among his patients fell to 17.3 per cent. —— Betz regards nitrate of silver as the best remedy for bed-sores. Instead of making use, however, of lint dipped in the solution of lunar caustic, he prescribes an ointment composed of five decigrammes of the nitrate of silver, fifteen grammes of lard, and thirty of wax; which he spreads on linen, and applies to the sores, taking care that the piece is rather larger than the sore. This is repeated morning and evening. —— Dr. George D. Hodge remarks in the *Georgia Med. Companion* that our best iron compounds are those having the *red oxide* as their base; and especially is *this* true when our aim is the restoration of impoverished blood. In *this* consists the efficacy of most mineral waters, as well as the advantages of the oxysulphates, over many others. The first formula is no *novelty*, but was used by Sylvester sixty years ago. It is: R. Crystallized Sulphate Iron, dr. ijss; Nitric Acid, dr. iij; Pure water, oz. iss. Stir well the iron and acid, by constant rubbing in a glass mortar, for at least twenty minutes—gradually add the water, and filter through white filtering paper. The result is a clear limpid fluid, which may be given in doses of from five to ten or fifteen drops, twice daily, in a little water or infusion of quassia—is easily prepared, and will be found, for general use, preferable to the muriated tincture of the shops—harmonizes chemically with, and confers solubility on quinine, sulph. magnesia, etc., and may be relied on as one of the very best restoratives for debility and torpor of the liver, following successfully treated cases of hepatitis or miasmatic fevers, in which the biliary organs have suffered. In malarial diseases, Dr. Hodge recommends: R. Sulph. Quinine, grs. xxx; Liq. Oxysulph. Iron, dr. j; Fowler's Sol., dr. j; Water, oz. ij. M. S. A teaspoonful thrice daily, after meals, occasionally adding a small dose of sulph. mag. to obviate costiveness. The iron and arsenic may be varied to meet the leading indication: R. Spts. Mindererus, oz. ij; Liq. Oxysulph. Iron, dr. j. M. This gives a beautiful florid mixture—

palatable, and in doses of a teaspoonful or so, thrice a day, not apt to disappoint as a general tonic. The ammonia promotes the action of the iron, and imparts to it a decided *capillary and renal tendency*. We have found the liquor oxysulph. in doses of from three to six minims with a drachm or two of the liquor ammonia acetat. every three or four hours, a valuable remedial agent in combating the forming stages of tonsilitis, scarlatina, typhoid fever, and puerperal inflammations. In these affections, I am convinced of the efficacy of this combination as superior to the mur. tinct. used in the same way. R. Iod. Pot., grs. xxx; Liq. Oxysulph. Iron, dr. ij; Simple Syrup, oz. j and dr. ij. M. Dissolve the iodide in the syrup and add the iron. This syrup is of a beautiful red color—will keep without deposition for years, and is a *most excellent tonic and alterative* in doses of 20 to 60 drops twice a day. We will close by adding, if the brom. pot. or brom. ammonia be substituted for the iodide (correspondingly as to quantity) in the above formula, a rich golden-colored compound will result, and prove itself on trial a remedy of the first class in all anæmic, chlorotic and nervous conditions resulting from uterine affections or otherwise. —— It is reported, says the *Medical and Surgical Reporter*, that distillers are experimenting with a process for making spirits from fermented garbage. This repulsive matter is placed in water-tight vats and boiled for several hours, the grease is then carefully skimmed off for soap-making purposes, and the remaining mass is fermented and distilled. The refuse is used as manure. It is stated that a barrel of garbage yields three pounds of soap grease and four gallons of proof spirits. —— Dr. Cowran, in his *Medical History of the Himalayas*, speaking of a native tribe in the northern district of the peninsula, says, when a mother goes into a field to work, or is otherwise unable to take her child with her, she selects some sheltered spot near a stream, in which she places a little straw for a bed for her infant, and then directs, by means of a piece of split bamboo, a current of water, of from one to two or three inches in diameter on its uncovered occiput and temples. This produces a soporific effect, which generally lasts as long as the water continues to flow. The sleep is said to be very soothing, and children who have been much subjected to its influence are known to have been unusually free from the annoyances incidental to the period of dentition. —— At the Central Medical Society of

New York, Dr. Weed lately presented a paper on the treatment of hæmorrhage of the bowels in typhoid fever, in which he referred to the grave complication of this hæmorrhage and its cure. It might be affirmed that in an exhaustive fever this symptom was an alarming one. It had occurred even in convalescence; various astringents had been recommended, but their operations were not always satisfactory. He gave the history of a case where blood was passing largely, and the prognosis was most unfavorable. The styptic properties of the oil of turpentine occurred to him, and he resolved to give it a trial; he gave teaspoonful doses repeated twice in thirty minutes, and then in smaller quantities, as the cases seemed to require; several other cases of a similar and very severe character, in which turpentine had always been given with *complete success*, were related. —— Dr. Eldridge says he has invariably cured mumps, even when *orchitis* had taken place, with an emetic, in twelve, fourteen or twenty-four hours! He never knew the emetic to fail! —— Dr. Moore, at the last session of the New York Med. Society, related a case where the condition of *albuminuria* was removed by the cathartic treatment. He had previously advocated cathartics, especially Epsom salts. Dr. Benedict gave the history of a case of similar character; the patient had been going rapidly downward, and he had almost given her up, when he heard the recommendation of Dr. Moore, and immediately gave her the remedy, and found relief from her dropsy and the difficulty of breathing she had experienced. In the course of six or seven days the dropsy had ceased, and the traces of albumen were less marked. She omitted the salts for a time, and was taken care of by a physician of Utica, who treated her differently. Her symptoms returned, and again Dr. Benedict treated with the salts, and found marked relief, but she was finally worn out and passed away. Dr. Campbell had two cases, one of which had been treated after Dr. Moore's plan. Sulphate of magnesia he thought had driven away the presence of albumen, but severe headache had continued; in the seventh month she had been taken with convulsions; he had put her under the influence of chloroform for three or four hours, and he had given all the sulphate of magnesia she could take; she continued through the day unconscious and no convulsions, and the next day seemed to be improving, but some shocks occurred. The test showed two-thirds albumen; coma occurred; one hand and foot were not used by the patient; artificial delivery followed, when she sank and died in a comatose condition. Dr. Mowris inquired whether this treatment had been successful after scarlet fever, and Dr. Moore replied in the affirmative. —— Dr. Byrd, Professor of Obstetrics in Washington University, once more raises the banner of bleeding, and says the practice is more satisfactory than any other. Indeed, he asserts in the *Medical and Surgical Reporter* that in *all* inflammatory diseases it is the most scientific and efficient agent, and that the necessity for it is as great at the present time as ever it was in the past. In certain conditions of the brain found in most cerebral diseases it is, we think, not only indispensable, but the only remedy necessary. —— In one of the papers on "Phthisis Pulmonalis," which recently appeared in the columns of the *Medical Press and Circular*, Dr. R. Locke Johnson speaks of the *increased tendency* of patients to hæmoptysis, during the spring time and the early summer time. Having for some years past made observations on this point, he has occasionally been enabled to retard altogether or to lessen this lesion, by attending closely to the secretions, the skin, and the pulse of his patients. —— At a recent meeting of the Societe de Chirurgie, Paris, M. Trelat related (*British Medical Journal*) a case of sudden death caused by the intrusion of air into the veins. M. Trelat was in the act of removing a submaxillary tumor when suddenly the patient changed color and the heart sounds ceased. Artificial respiration and electrization of the phrenic nerve induced a few respiratory movements, and a slight return of color appeared after this treatment had been persisted in for fifteen minutes. The necropsy revealed the partial division of a small vein which opened into the external jugular. There was an oblong clot in the jugular segmented by air blebs. Bubbles of air were also found in one of the mediastinal veins, the posterior cardiac vein, and a notable quantity was also found in the right cardiac cavities. Chloroform had been administered, so the question raised was if death might not be attributed to that drug. M. Perrin and M. Marc See considered such to be the case. M. Giraldes believed entrance of air into the veins acting with the chloroform produced the fatal result, and stated that in three cases of death from chloroform he found the presence of gases in the heart of each case. In the vena cava, and even in the veins of the pelvis, M. Roux had made a similar observation, but M. Depaul pointed out that the air in this case occupied only the veins going to the heart and the wounded vein. The employment of artificial respiration by means of a tracheal catheter and bellows was advocated by M. M. Perrin and Depaul. —— Dr. Grimshaw (*British Medical Journal*) states that he considers *pressure* is the main point to be relied on—by lessening the vascularity of the part and preventing the maturation of the pustules—in cases where pitting from small-pox is feared. Strapping, in parts where it may be possible to do so, answers this purpose fully, and the next best thing to strapping is to employ xylonite, a collodion which, although somewhat elastic, quickly forms—when employed over the cuticle—a dense and tight skin, more dense, tight and tenacious than the collodion of the *Pharmacopœia*. It acts like strapping, and produces abortion of the pustules. To parts of the face where strapping cannot be employed with advantage, xylonite will be found to be the next best mode of treatment. The late Dr. Graves remarked that in a patient whose knee had been strapped for a surgical operation being attacked by small-pox, the portion over which the strapping extended was free from even the rash. —— At a recent meeting of the Pathological Society of London, Dr. Thorowgood exhibited for Dr. R. Locke Johnson, two gall stones removed from a female patient æt. 67, and who had suffered no inconvenience from their presence during her lifetime. They completely occluded the gall bladder, assumed its shape, were composed principally of cholesterine, and weighed at the period of removal 580 grains, the larger one weighing 420 grains. Probably these are the largest gall stones ever removed from a female patient. —— M. M. Mouttet and Rosiere, of Montpelier, report that they have cured croup, when tracheotomy was refused by the parents as a useless cruelty, by repeated insufflations of powdered nitrate of silver. The application was at once followed by the detachment and expulsion of false membrane, and was repeated a quarter of an hour after with a similar result. Three or four hours later another insufflation was followed by so much relief that hope was entertained, and the child from this time made a steady recovery. —— Dr. Wilks, of Guy's, in a paper in the *Lancet*, indorses the conclusions lately published by Dr. Handfield Jones, in reference to the production of epilepsy and other nervous diseases by the abuse of alcohol. Dr. Wilks has met with cases where the alcohol, acting chiefly on the spinal cord, made paralysis the leading symptom, and he combats, in his own trenchant style, the notion that it is not safe to suddenly leave off the accustomed stimulus. "No harm," he says, "but only good will ensue from its withdrawal." He considers that the same rule should apply to all persons. Some striking cases are mentioned, in which the absolute and instant withdrawal of alcohol snatched the patients from the very jaws of death. One was a professional man, who, after drinking hard, became so ill that he took to his bed, had epileptiform attacks, ate nothing, and was constantly retching, his wife standing over him administering brandy and champagne from time to time to keep him alive a little longer. Dr. Wilks succeeded, after several attempts, in inducing his wife and two medical attendants to stop every drop of alcohol. When this was done, the patient soon cried out for drink; but, after imploring in vain for some time, he was violently sick, and then sank into a sound sleep. Upon waking he took a little beef-tea, in a few hours ate some solid food, and within a week was back again in his practice. The purport of Dr. Wilks's paper is to draw attention to the fact of paralysis, and more particularly spinal paralysis, occurring as a result of alcoholism; and therefore that when a medical man is called in to see a case of this kind, he should remember intemperance in drinking as a possible cause, just as he would if he found an enlarged liver. If the affection should turn out to be in any way peculiar in its pathology it will certainly deserve a distinct appellation; but even should the morbid changes in the cord, together with the resulting symptoms, resemble what is seen in other forms of paralysis, he would still recommend the adoption of such a term as alcoholic paralysis as significant of its cause, for we are warranted in so doing by the use of the expression puerperal, syphilitic, or uræmic epilepsy (eclampsia) in reference to the origin of the fits when arising under special circumstances. —— In an abstract of a clinical lecture at Cambridge, on the early diagnosis of typhoid, published in the *Lancet*, Dr. P. W. Latham insists on the value of the thermometer, observing that during the first four or five days the general symptoms which may then accompany the disease—viz., the rigor, the languor and feebleness, headache, epistaxis, giddiness, pain in the back and aching of the limbs, the appearance of the tongue, the state of the bowels, the condition of the urine, etc.—may not be very distinct, or any one of these morbid symptoms may be entirely absent. In a considerable number of cases, in fact, it would be impossible to say, without using the thermometer, whether the patient were suffering from typhoid fever or not. But the thermometric course of the disease at this time, unless it supervenes on some other malady, is very regular; and by taking the temperature at 8 A. M. and 6 P. M. for three days the presence of typhoid fever may be decided. On the other hand, one single observation may, with very great probability, negative the existence of the disease. The following is the formula (from Wunderlich) of this initial stage: 1st day, morning, 98.6° F., evening, 100.4° F.; 2nd day, morning, 99.4° F., evening, 101.4° F.; 3rd day, morning, 100.4° F., evening, 102.6° F.; 4th day, morning, 101.6° F., evening, 104° F. If, then, a person, previously quite well, feels uneasy, perhaps has a rigor, and in the evening we find his temperature about 100.4° or 101° F., falling the next morning about a degree, rising again in the evening, and approximately following the above course, the disease may be diagnosed with tolerable certainty. On the other hand, the disease is not typhoid fever if (1) on the second, third or fourth evenings the temperature approximates even to normal (98.6° F.); (2) if during the first two days the temperature rises to 104° F.; (3) if between the fourth and sixth days the evening temperature of a person under middle age does not reach 103°; (4) if the temperature on two of the first three evenings is the same; or (5) if it is the same on the second and third mornings. From the fourth to the tenth day the evening temperatures are tolerably uniform, the highest being most generally on the evenings of the fourth, fifth or sixth days, and reaching from 104° to 105.5° F., or even higher. The morning temperatures are from 1° to 2.6° F. lower than the evening ones; on the fifth, sixth and seventh days, the variations between the morning and evening temperatures being less than take place from the sixth or seventh to the ninth or tenth days. During this period (from the fourth to the tenth or twelfth day), if the general symptoms are obscure, an absolute diagnosis may not be readily made, and the disease may be confounded with several others, unless thermometric observations extend over several days.

